# Cross-Species Conservation and Function of ALDH6A1/Aldh6a1 Validate Zebrafish and Mouse as Complementary Experimental Systems for Methylmalonate Semialdehyde Dehydrogenase Deficiency

**DOI:** 10.3390/biology15161345

**Published:** 2026-08-08

**Authors:** Yanping Zhang, Xiaoqiao Yue, Qiuhong Xiong, Ping Li, Changxin Wu

**Affiliations:** 1 Key Laboratory of Molecular Cell Medicine of Shanxi Province, Institutes of Biomedical Sciences, Shanxi University, Taiyuan 030006, China; 201923105006@email.sxu.edu.cn (Y.Z.); qxiong@sxu.edu.cn (Q.X.); 2 Biomedicinal and Health Laboratory in Shanxi Province, Shanxi University, Taiyuan 030006, China; 3 Shanxi Bethune Hospital (Shanxi Academy of Medical Sciences, Third Hospital of Shanxi Medical University, Tongji Shanxi Hospital), Taiyuan 030032, China; yuexiaoqiao@sxbqeh.com.cn; 4 Key Laboratory of Chemical Biology and Molecular Engineering of Education Ministry, Shanxi University, Taiyuan 030006, China

**Keywords:** ALDH6A1/Aldh6a1, evolutionary conservation, spatiotemporal expression, zebrafish, mouse, MMSDD

## Abstract

Methylmalonate semialdehyde dehydrogenase deficiency (MMSDD; OMIM #614265) is an ultra-rare autosomal recessive metabolic disorder caused by mutations in the *ALDH6A1* gene, characterized by multi-system involvement including skeletal abnormalities, hypotonia, and visual impairment. To elucidate its pathogenic mechanisms, we characterized ALDH6A1/Aldh6a1 conservation and function using complementary zebrafish and mouse experimental systems. Bioinformatic and phylogenetic analyses revealed strong conservation of *Aldh6a1/aldh6a1* gene structure, protein sequence, functional domains, and mitochondrial targeting across vertebrates. Spatiotemporal profiling also demonstrated dynamic embryonic expression in zebrafish neural tube, somites, liver, and intestine, as well as in metabolically active and developmentally relevant tissues of mice, with patterns that correspond to known MMSDD lesion sites in humans. Functional disruption of Aldh6a1 in zebrafish induced dose-dependent developmental defects, including spinal curvature, tail coiling, pericardial edema, and reduced survival, which were partially rescued by exogenous mRNA, confirming phenotype specificity effects. These findings support a role for ALDH6A1 in mitochondrial metabolism during neurogenesis, myogenesis, and organogenesis. Collectively, this cross-species framework highlights ALDH6A1/Aldh6a1 as essential for vertebrate development, provides mechanistic insight into the multi-system heterogeneity of MMSDD, and establishes a scalable platform for therapeutic discovery and precision intervention.

## Simple Summary

Methylmalonate semialdehyde dehydrogenase deficiency (MMSDD; OMIM #614265) is an extremely rare inherited metabolic disorder caused by mutations in the *ALDH6A1* gene. Patients with MMSDD often suffer from severe developmental delay, muscle weakness, and neurological impairment, yet the underlying disease mechanisms remain poorly understood due to a lack of suitable animal models. In this study, we systematically compared the *Aldh6a1/aldh6a1* gene and protein across multiple species and demonstrated that both mice and zebrafish possess highly conserved *Aldh6a1/aldh6a1* orthologs that closely resemble the human counterpart. We further characterized the tissue distribution and embryonic expression patterns of ALDH6A1/Aldh6a1 in both species, and functionally validated that zebrafish with reduced Aldh6a1 levels recapitulate key disease-associated phenotypes. Our findings establish mice and zebrafish as valuable experimental systems for MMSDD, providing essential tools for future studies aimed at unraveling the pathogenesis of this disorder and developing potential therapeutic strategies.

## 1. Introduction

Methylmalonate semialdehyde dehydrogenase deficiency (MMSDD; OMIM #614265) is an extremely rare autosomal recessive disorder, with only five cases reported worldwide to date [1]. The disease exhibits considerable clinical heterogeneity, primarily characterized by developmental delay, hypomyelination, and hypotonia. Additional clinical manifestations, including hepatosplenomegaly and gastroesophageal reflux, have also been observed in certain patients, severely compromising their quality of life and overall prognosis [1,2,3,4,5]. However, due to the extreme rarity of the disease, current understanding of its pathogenesis is largely limited to the effects of mutations on protein structure and function [1,2,3,4,5], and effective therapeutic strategies remain lacking. Therefore, the establishment of reliable animal models to elucidate the underlying molecular mechanisms of its pathology has become an urgent and critical task in this field.

Cellular catabolism comprises a series of biochemical pathways that convert nutrients into metabolic intermediates, thereby supplying energy for essential biological processes including cell growth and proliferation [6,7,8]. During this process, aldehydes are generated as key intermediate metabolites from the catabolism of glucose, fatty acids, amino acids, and exogenous alcohols [9,10,11]. Owing to their high reactivity and strong electrophilicity, aberrant accumulation of aldehydes within the cell leads to the formation of adducts with proteins, DNA, and RNA, resulting in enzyme inactivation, metabolic disruption, DNA damage, and ultimately cell death [12,13,14]. Consequently, cells have evolved a sophisticated detoxification system for aldehyde metabolism, within which the aldehyde dehydrogenase (ALDH) family plays a central role [15].

Aldehyde dehydrogenases (ALDHs) constitute a superfamily of NAD(P)+-dependent enzymes characterized by conserved primary structures and molecular weights typically ranging from 50 to 60 kDa [11]. These enzymes catalyze the oxidation of diverse endogenous and exogenous aliphatic and aromatic aldehydes to their corresponding carboxylic acids, thereby contributing to cellular detoxification [16,17]. Individual ALDH isoforms exhibit distinct subcellular localization, tissue distribution, and substrate specificity, and collectively maintain metabolic homeostasis through enzymatic activity [18,19,20]. ALDH6A1 is the sole CoA-dependent enzyme within the ALDH superfamily. It participates in the degradation pathways of valine, leucine, isoleucine, and pyrimidines, catalyzing the conversion of methylmalonate semialdehyde to acetyl-CoA and propionyl-CoA [18]. Therefore, a comprehensive investigation of the biological functions of ALDH6A1 and its spatiotemporal expression patterns during embryonic development is of critical importance for understanding the pathogenic mechanisms underlying MMSDD.

Mice and zebrafish offer distinct advantages as model organisms in developmental biology and genetic research [21,22,23]. Mice share high genomic homology, conserved metabolic pathways, and similar organ system architecture with humans, making them particularly suitable for metabolic phenotyping and therapeutic validation. Zebrafish, on the other hand, are characterized by external fertilization, embryonic transparency, and a short reproductive cycle, rendering them an ideal platform for high-throughput drug screening and studies of early developmental mechanisms. However, systematic investigations into the expression profiles, subcellular localization, and functional conservation of ALDH6A1/Aldh6a1 in these two species remain scarce, limiting cross-species validation of ALDH6A1/Aldh6a1 function and its relevance to MMSDD pathogenesis.

In this study, we systematically characterized ALDH6A1/Aldh6a1 conservation and function across both species to evaluate the suitability of zebrafish and mouse experimental systems for MMSDD research. Using subcellular localization, spatiotemporal expression profiling, and loss-of-function rescue in zebrafish, we found that the expression dynamics of ALDH6A1/Aldh6a1 orthologs closely mirrored the organ systems affected in human MMSDD patients. These findings provide critical insights into the molecular pathogenesis of the disease and establishing a robust platform for future mechanistic studies.

## 2. Materials and Methods

### 2.1. Animal Husbandry

AB strain zebrafish (*Danio rerio*) were maintained at 28 °C on a 14 h/10 h light/dark cycle with twice-daily feeding. Embryos were obtained by natural spawning, incubated at 28.5 °C in E3 medium (5 mM NaCl, 0.17 mM KCl, 0.33 mM CaCl_2_·2H_2_O, 0.33 mM MgSO_4_·7H_2_O) [24,25], and staged according to established criteria. C57BL/6 mice (*Mus musculus*) were housed under specific pathogen-free (SPF) conditions at 22 ± 2 °C and 50 ± 10% relative humidity on a 12 h/12 h light/dark cycle, with irradiated standard chow and autoclaved water ad libitum. All procedures were approved by the Institutional Animal Care and Use Committee.

### 2.2. Bioinformatics Analysis

Nucleotide and amino acid sequences were retrieved from Ensembl (https://www.ensembl.org/index.html?redirect=no), URL (accessed on 15 April 2026). Gene structures were visualized using the Gene Structure Display Server (GSDS, https://gsds.gao-lab.org/Gsds_help.php), URL (accessed on 17 April 2026) [26]. Chromosomal synteny was analyzed across human, mouse and zebrafish loci. Phylogenetic analysis of 19 vertebrate ALDH6A1/Aldh6a1 orthologs was performed using MEGA 11.0 (Neighbor-Joining method) [27]. Sequence alignment of human, mouse, and zebrafish ALDH6A1/Aldh6a1 was conducted with ClustalX [28]. Three-dimensional structures from UniProt were visualized in PyMOL (version 2.6.0) [29].

### 2.3. Molecular Cloning and Plasmid Construction

Primers targeting the coding sequence (CDS) regions of human, mouse, and zebrafish *aldh6a1* genes were designed and synthesized as follows: h*ALDH6A1*-Fw: 5′-ATGGCGGCGCTATTGGCGGC-3′; h*ALDH6A1*-Rv: 5′-ACGGCCCATGGTAGGCATGACAAC-3′; m*Aldh6a1*-Fw: 5′-ATGGCGGCGGCAGTGGC-3′; m *Aldh6a1*-Rv: 5′-ACGCCCCATGGTAGGCATGA-3′; D-*aldh6a1*-Fw: 5′-ATGGCAGCGATATTAGTGCGT-3′; D-*aldh6a1*-Rv: 5′-ACGCCCCATTGTTGGCATG-3′. PCR products were cloned into linearized pCS2-GFP using a one-step cloning kit and verified by Sanger sequencing (Sangon Biotech, Shanghai, China).

### 2.4. Cell Culture and Transfection

HEK293T cells were cultured in high-glucose DMEM (Solarbio, Beijing, China, Cat. No.11995) supplemented with 10% (*v*/*v*) FBS (Servicebio, Wuhan, China, Cat. No. G8003) and 100 U/mL penicillin–streptomycin (Solarbio, Beijing, China, Cat. No.P1400) at 37 °C with 5% CO_2_. Transfection was performed using polyethylenimine (PEI; Polysciences, Warrington, PA, USA, Cat. No. 23966-2) according to the manufacturer’s protocol. Cells were harvested 24–48 h post-transfection.

### 2.5. Immunofluorescence

Cells on coverslips were fixed in 4% paraformaldehyde for 10 min, washed with PBS, and stained with DAPI (Solarbio, Beijing, China, Cat. No. ID2250). Images were acquired on a confocal microscope (63× objective).

### 2.6. Western Blot

Cells were lysed in RIPA buffer with 1 mM PMSF, sonicated, and centrifuged at 12,000× *g* for 10 min at 4 °C. Supernatants were mixed with 5× SDS loading buffer, boiled (95 °C, 10 min), and re-centrifuged. Proteins were separated by 10% SDS-PAGE (120 V, 1.5 h) and transferred to membranes (300 mA, 2 h, 4 °C). Membranes were blocked in 5% non-fat milk/TBST (1 h, RT), incubated with primary antibodies (ALDH6A1, Proteintech, Wuhan, China, 20452-1-AP; ACTIN, Proteintech, Wuhan, China, 66009-1-Ig) overnight, washed with TBST (3×), incubated with secondary antibodies (HRP-conjugated goat anti-rabbit IgG (H+L)—Proteintech, Wuhan, China, SA00001-2; HRP-conjugated goat anti-mouse IgG (H+L)—Proteintech, Wuhan, China, SA00001-1) (1 h, RT), washed again (3×, 10 min each), and visualized by chemiluminescence.

### 2.7. Whole-Mount In Situ Hybridization

Zebrafish *aldh6a1* fragments were amplified (primers: 5′-ATGGCAGCGATATTAGTGCGT-3′ and 5′-GTGTTTCCGCACACCATGCC-3′) and cloned into pUCm-T (Promega, Madison, WI, USA). DIG-labeled antisense probes were synthesized by in vitro transcription (Roche, Basel, Switzerland). Embryos were fixed, treated with 0.003% phenylthiourea (PTU; Sigma-Aldrich, St. Louis, MO, USA) before 24 hpf [30,31], hybridized with probes, incubated with anti-DIG antibody (Roche, Basel, Switzerland), and developed with NBT/BCIP (Roche, Basel, Switzerland). Images were captured on an OLYMPUS stereomicroscope (Olympus, Tokyo, Japan).

### 2.8. Morpholino Microinjection and Rescue Experiments

Splice-blocking morpholino oligonucleotides (spl-MO) targeting the exon5-intron6 boundary of zebrafish *aldh6a1* (sequence: 5′-TGTGTAATTTTCCATACCGTCATGC-3′; Gene Tools, LLC; Philomath, OR, USA) were designed to interfere with pre-mRNA splicing. A standard control morpholino was used as a negative control. Morpholinos were resuspended in sterile water to a stock concentration of 1 mM and diluted to working concentrations (0.3 mM and 0.5 mM) in 1× Danieau buffer containing 0.05% phenol red as a tracer. Approximately 1–2 nL of morpholino solution was microinjected into the yolk of one- to two-cell stage embryos using a pneumatic microinjector. For rescue experiments, capped *aldh6a1* mRNA was synthesized using the mMESSAGE mMACHINE SP6 kit (Thermo Fisher, Thermo Fisher Scientific, Waltham, MA, USA) from pCS2-*aldh6a1* linearized with NotI, and co-injected with spl-MO at a concentration of 100 ng/μL. Embryos were incubated at 28.5 °C and examined at specified developmental stages.

### 2.9. Quantitative RT-PCR

Total RNA was extracted with Trizol and reverse-transcribed. qPCR was performed on a CFX Connect system (Bio-Rad, Hercules, CA, USA) using the following primers: q-*aldh6a1*-F: 5′-ACCAGGCTGGCGAGTACATC-3′; q-*aldh6a1*-R: 5′-TCCAAACGCAGCACCTACCA-3′; q-*Rpl13α*-F: 5′-TCTGGAGGACTGTAAGAGGTATGC-3′; q-*Rpl13α*-R: 5′-GTCTTTCCATGTGACCTCCCTTC-3′; q-*18S rRNA*-F: 5′-AAGAGCGTAATAGGCACCAGTTC-3′; q-*18S rRNA*-R: 5′-GTTCTCCCAGCCCATTCTCTC-3′. All reactions were performed in accordance with standard protocols.

### 2.10. Immunohistochemistry (IHC)

Pregnant C57BL/6J mice were sacrificed at embryonic day 16.5 (E16.5), and embryos were isolated and freed of fetal membranes. Whole embryos were fixed overnight in 4% paraformaldehyde. Subsequent trimming, paraffin embedding, and sagittal sectioning (4 μm thickness) were performed by Wuhan Servicebio Technology Co., Ltd. (Servicebio, Wuhan, China). After deparaffinization, rehydration, and antigen retrieval, HE staining and immunohistochemistry were carried out by the same company. For IHC, sections were incubated overnight at 4 °C with rabbit anti-ALDH6A1 polyclonal antibody (Proteintech, Wuhan, China; 1:300 dilution, Cat. No. 20452-1-AP) and rabbit anti-DESMIN monoclonal antibody (Servicebio, Wuhan, China; 1:500 dilution, Cat. No. GB15075-100). Immunoreactivity was visualized using DAB substrate, followed by counterstaining with hematoxylin. After dehydration and clearing, the sections were coverslipped and scanned using a whole-slide scanner (Olympus, Tokyo, Japan) for digital image acquisition and analysis.

### 2.11. Cross-Species Comparison Criteria

To enable systematic comparison between zebrafish and mouse experimental systems, equivalent developmental stages and anatomical systems were aligned based on conserved MMSDD-relevant phenotypes. In zebrafish, developmental stages from somitogenesis (4–15 ss) through 96 hpf were selected to cover neural tube formation, somitogenesis, and early organogenesis. In mice, embryonic day 16.5 (E16.5) was chosen as a comparable developmental window for major organ system differentiation. Cross-species comparison of ALDH6A1/Aldh6a1 expression was performed as follows: in zebrafish, spatial distribution was determined by whole-mount in situ hybridization using an *aldh6a1*-specific antisense probe, with anatomical identification based on established developmental landmarks and morphological criteria. In mice, tissue distribution was determined by immunohistochemistry using an anti-ALDH6A1 antibody, with tissue and cell-type identification supported by adjacent hematoxylin and eosin (H&E) stained sections and standard histological morphology. For skeletal muscle characterization, DESMIN immunostaining was used as a complementary marker.

## 3. Results

### 3.1. Bioinformatics Analysis of ALDH6A1/Aldh6a1

**Figure 1:**
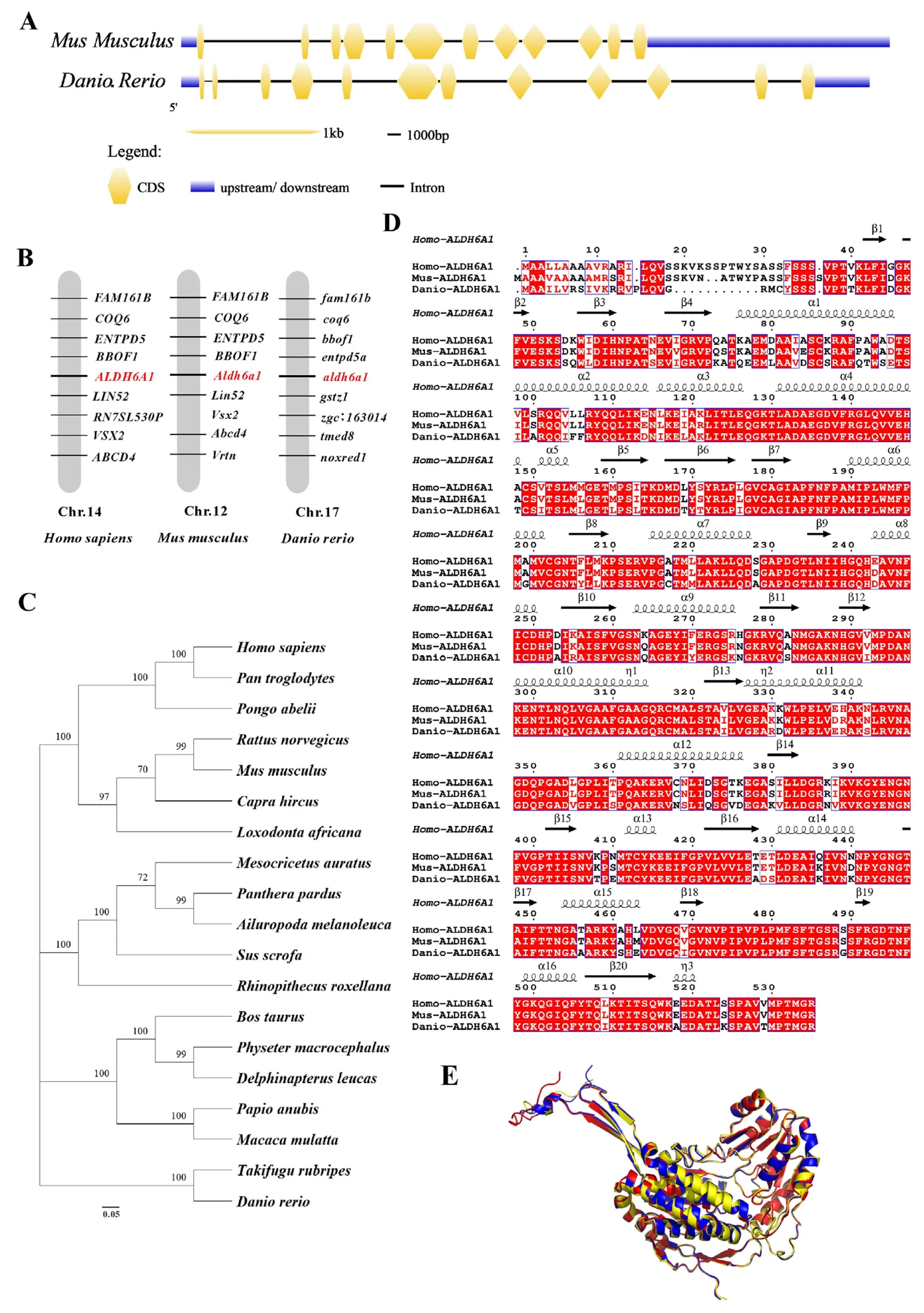
Bioinformatic analysis of *Aldh6a1/aldh6a1* gene and protein in mouse and zebrafish. (**A**) Gene structures of *Aldh6a1/aldh6a1* in Mus musculus and Danio rerio. Gene sequences were obtained from the Ensembl database, and structural schematics were generated using GSDS. (**B**) Chromosomal synteny analysis of ALDH6A1/ALDH6A1/Aldh6a1 among human, mouse, and zebrafish. (**C**) Phylogenetic tree of ALDH6A1/Aldh6a1 constructed from amino acid sequences of 19 vertebrate species using the Neighbor-Joining method implemented in MEGA11. Accession numbers are as follows: Homo sapiens (NP_005580.1), Pan troglodytes (XP_009429713.1), Pongo abelii (XP_002814416.1), Rattus norvegicus (NP_001100628.1), Mus musculus (NP_038556.2), Capra hircus (XP_005679590.1), Loxodonta africana (XP_003419110.1), Mesocricetus auratus (XP_005074934.1), Panthera pardus (XP_053745564.1), Ailuropoda melanoleuca (XP_002914734.1), Sus scrofa (XP_005658744.1), Rhinopithecus roxellana (XP_010777116.1), Bos taurus (XP_005227369.1), Physeter macrocephalus (NP_001297124.1), Delphinapterus leucas (XP_022437776.1), Papio anubis (XP_003911706.1), Macaca mulatta (NP_001180799.1), Takifugu rubripes (XP_003966956.1), and Danio rerio (NP_001003581.1). (**D**) Multiple sequence alignment of ALDH6A1 amino acid sequences from human, mouse, and zebrafish performed using ClustalX. Identical residues are shaded in red, and similar residues are boxed in blue. Numbers and dots above the alignment indicate residue positions marked at every tenth amino acid for orientation. Secondary structure elements derived from the human ALDH6A1 crystal structure (PDB: 8XXQ) are indicated above the alignment: α-helices are shown as coiled springs and β-strands as arrows. (**E**) Comparative analysis of the tertiary structures of human, mouse, and zebrafish ALDH6A1/ALDH6A1/Aldh6a1 proteins, visualized using PyMOL. Human: blue; mouse: red; zebrafish: yellow.

To evaluate the gene structure of *Aldh6a1/aldh6a1* in mice and zebrafish, gene models were generated using GSDS. The zebrafish *aldh6a1* gene (NC_007128.7) spans approximately 30 kb and comprises 12 exons, producing a single transcript (NM_001002374.2) encoding a 524-amino-acid protein. The full-length cDNA is 2129 bp, including a 133 bp 5′ untranslated region (UTR), a 1578 bp open reading frame (ORF), and a 418 bp 3′UTR (Figure 1A). Similarly, the mouse *Aldh6a1* gene (NC_000078.6) spans approximately 30 kb and contains 12 exons. The longest transcript (NM_134042.3) encodes a 535-amino-acid protein, with a total cDNA length of 3420 bp, including an 85 bp 5′UTR, a 1608 bp ORF, and a 1727 bp 3′UTR (Figure 1A). Chromosomal synteny analysis across human, mouse, and zebrafish revealed highly conserved genomic linkage of zebrafish *aldh6a1* with four neighboring genes, suggesting evolutionary conservation with mammalian orthologs (Figure 1B). Phylogenetic analysis of ALDH6A1/Aldh6a1 amino acid sequences from multiple vertebrate species demonstrated that zebrafish Aldh6a1 is highly conserved and clusters with its orthologs from human and other vertebrates, supported by high bootstrap values (Figure 1C).

To assess the evolutionary conservation of ALDH6A1/Aldh6a1 across species, multiple sequence alignment was performed using ClustalX and GeneDoc. ALDH6A1/Aldh6a1 is highly conserved, showing 92% amino acid identity between human and mouse, and 84% between human and zebrafish (Figure 1D). Structural comparison of predicted zebrafish and mouse Aldh6a1/ALDH6A1 proteins with the experimentally determined human ALDH6A1 structure (PDB: 8XXQ) [32] revealed a high degree of structural similarity (Figure 1E). All three orthologs contain conserved functional domains, including a mitochondrial targeting sequence, an NAD-binding domain, an oligomerization domain, and a catalytic domain, indicating functional conservation across species. Together, these data demonstrate that ALDH6A1/Aldh6a1 has been subjected to strong selective pressure throughout vertebrate evolution, preserving not only gene structure and sequence but also the entire functional framework of the protein.

### 3.2. Analysis of ALDH6A1/Aldh6a1 Protein Expression and Subcellular Localization in Mouse and Zebrafish

**Figure 2:**
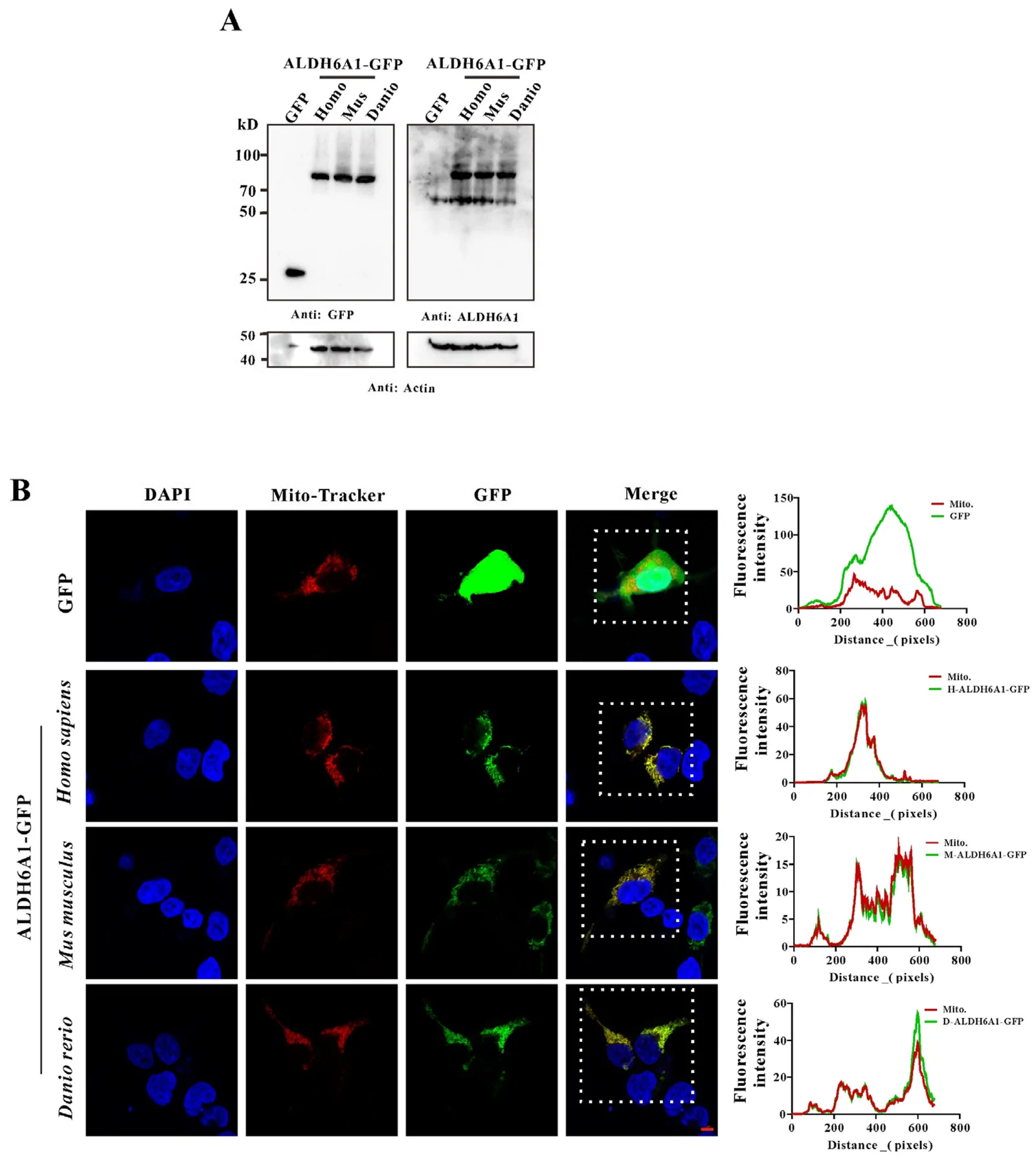
Expression and subcellular localization of ALDH6A1/ALDH6A1/Aldh6a1 proteins in human, mouse, and zebrafish. (**A**) Western blot analysis of heterologously expressed human, mouse, and zebrafish ALDH6A1/Aldh6a1-GFP fusion proteins. Proteins were detected using anti-GFP and anti-human ALDH6A1 antibodies, with β-actin used as a loading control. (**B**) Subcellular localization of ALDH6A1/Aldh6a1-GFP fusion proteins from human, mouse, and zebrafish examined by immunofluorescence microscopy. Green fluorescence represents GFP or ALDH6A1-GFP signals, red fluorescence indicates mitochondria, and DAPI marks nuclei with blue fluorescence. The white boxes indicate the regions used for colocalization analysis.

Given the high structural conservation of ALDH6A1/Aldh6a1 across species (Figure 1E), we next investigated whether this similarity extends to protein expression and subcellular localization. GFP-tagged expression constructs of human, mouse, and zebrafish ALDH6A1/ALDH6A1/Aldh6a1 were generated and transiently transfected into cultured cells. Western blot analysis confirmed successful overexpression of all three proteins, with electrophoretic mobilities consistent with their expected molecular weights (Figure 2A). To determine subcellular localization, immunofluorescence microscopy was performed following co-staining with MitoTracker. In cells expressing human ALDH6A1-GFP, the GFP signal extensively overlapped with MitoTracker staining, confirming its mitochondrial localization (Figure 2B). Notably, both mice and zebrafish ALDH6A1/Aldh6a1-GFP exhibited identical punctate patterns and precisely co-localized with MitoTracker in merged images (Figure 2B). Collectively, these findings demonstrate that mitochondrial targeting of ALDH6A1/Aldh6a1 has been conserved throughout evolution from fish to mammals, thereby defining the subcellular microenvironment in which pathogenic mutations exert their effects.

### 3.3. Spatiotemporal Expression of Aldh6a1 During Zebrafish Development

**Figure 3:**
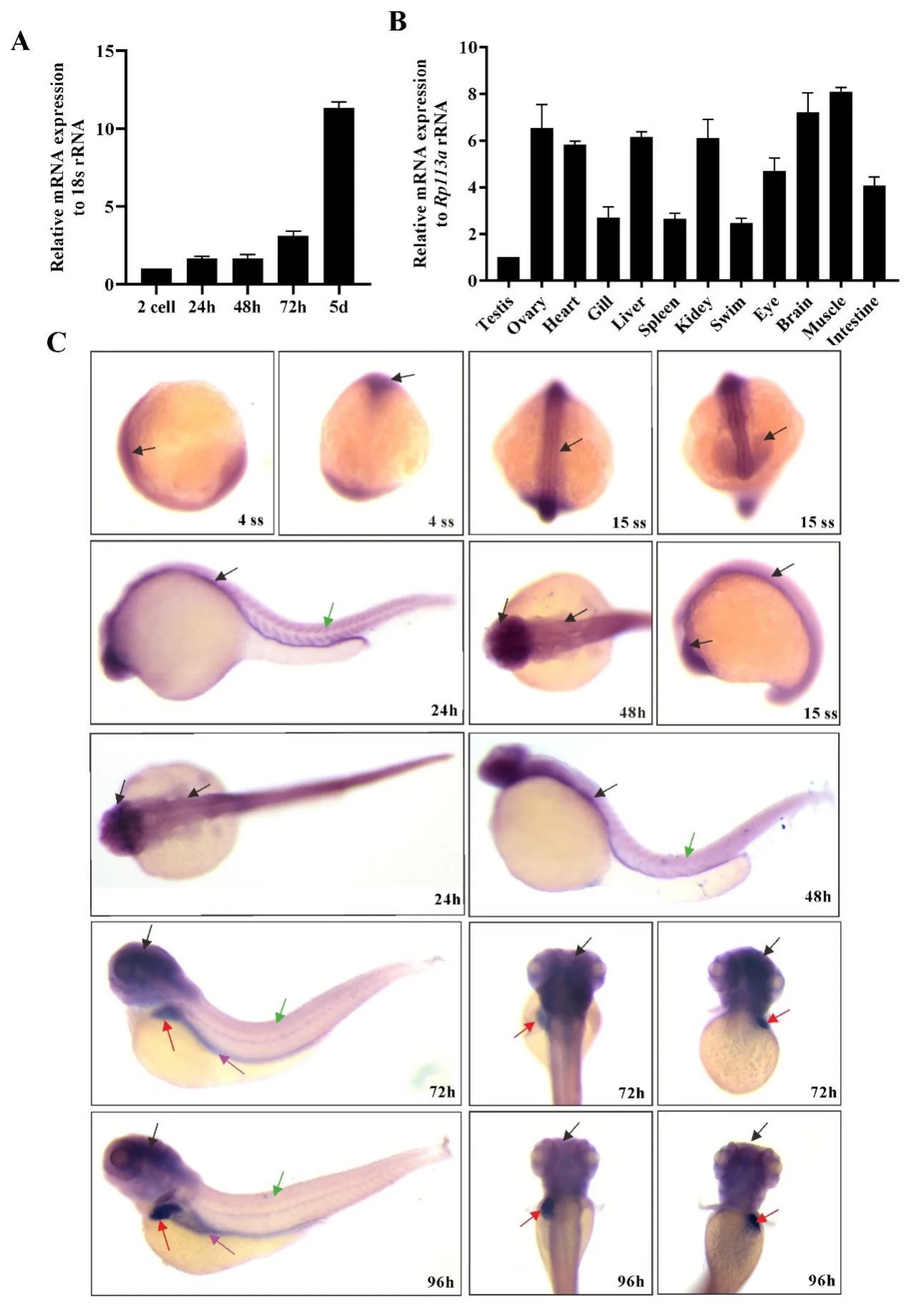
Spatiotemporal expression of *aldh6a1* in zebrafish. (**A**) Temporal expression profile of *aldh6a1* during zebrafish embryonic development from the 2-cell stage to 96h, quantified by qRT-PCR. Expression levels were normalized to the 2-cell stage. (**B**) Tissue-specific expression of *aldh6a1* in adult zebrafish assessed by qRT-PCR, with expression normalized to the testis. (**C**) Spatiotemporal expression pattern of *aldh6a1* in zebrafish embryos determined by whole-mount in situ hybridization. Black arrows indicate the nervous system; green arrows indicate muscle; red arrows indicate liver; purple arrows indicate the digestive system.

To evaluate spatiotemporal expression of *aldh6a1* during zebrafish development, qRT-PCR was performed on embryos at sequential developmental stages and relative expression levels were normalized to the 2-cell stage (Figure 3A). Maternal *aldh6a1* transcripts were detected at low levels and increased progressively during embryogenesis, with expression remaining stable between 24 and 48 hpf. Tissue-specific expression in adult zebrafish was also examined, with relative levels normalized to that in the testis (Figure 3B). Zebrafish *aldh6a1* mRNA was highly expressed in the heart, kidney, liver, brain, and muscle, consistent with the expression pattern observed in human tissues. Together, these results highlight the temporal and tissue-specific expression pattern of *aldh6a1* in zebrafish.

To further characterize the spatial expression of *aldh6a1* during zebrafish development, whole-mount in situ hybridization was performed using digoxigenin-labeled antisense RNA probes. A dynamic spatiotemporal expression pattern was observed throughout embryogenesis, with prominent expression during neural tube formation, somitogenesis, and organogenesis. At the 4-somite stage (4 ss), diffuse linear staining was observed along the dorsal midline, extending along the anterior–posterior axis and spanning the width of the neural plate. By the 15-somite stage (15 ss), the expression of *aldh6a1* mRNA became restricted to the neural tube, forming a distinct linear domain from the anterior region to the tail bud in lateral view and a symmetrical longitudinal stripe in dorsal view; no somitic expression was detected. From 24 to 48 hpf, the expression of *aldh6a1* mRNA expanded markedly, exhibiting a combined neuroectodermal and mesodermal pattern. Strong continuous signals extended throughout the central nervous system, while somites displayed a periodic, segmented stripe-like pattern. Intense staining in the head region indicated widespread expression in the developing brain. At 72 hpf, the expression pattern became more refined, with somite stripes appearing more regular and densely organized. New expression domains emerged in the digestive system, including moderate signals in the liver primordium and linear staining in the intestine. From 96 hpf, *aldh6a1* expression persisted in the central nervous system and muscle. Notably, strong signals were maintained in the liver, and expression in the intestine became increasingly prominent, indicating enhanced expression in the developing digestive system. Together, these spatiotemporal data demonstrate that zebrafish *aldh6a1* mRNA dynamically functions in tissues corresponding to the major lesion sites of MMSDD, namely the neural, muscular, hepatic, and intestinal systems, further validating the suitability of zebrafish as a developmental experimental system for this disease.

### 3.4. Tissue Distribution and Embryonic Expression of ALDH6A1 in Mice

**Figure 4:**
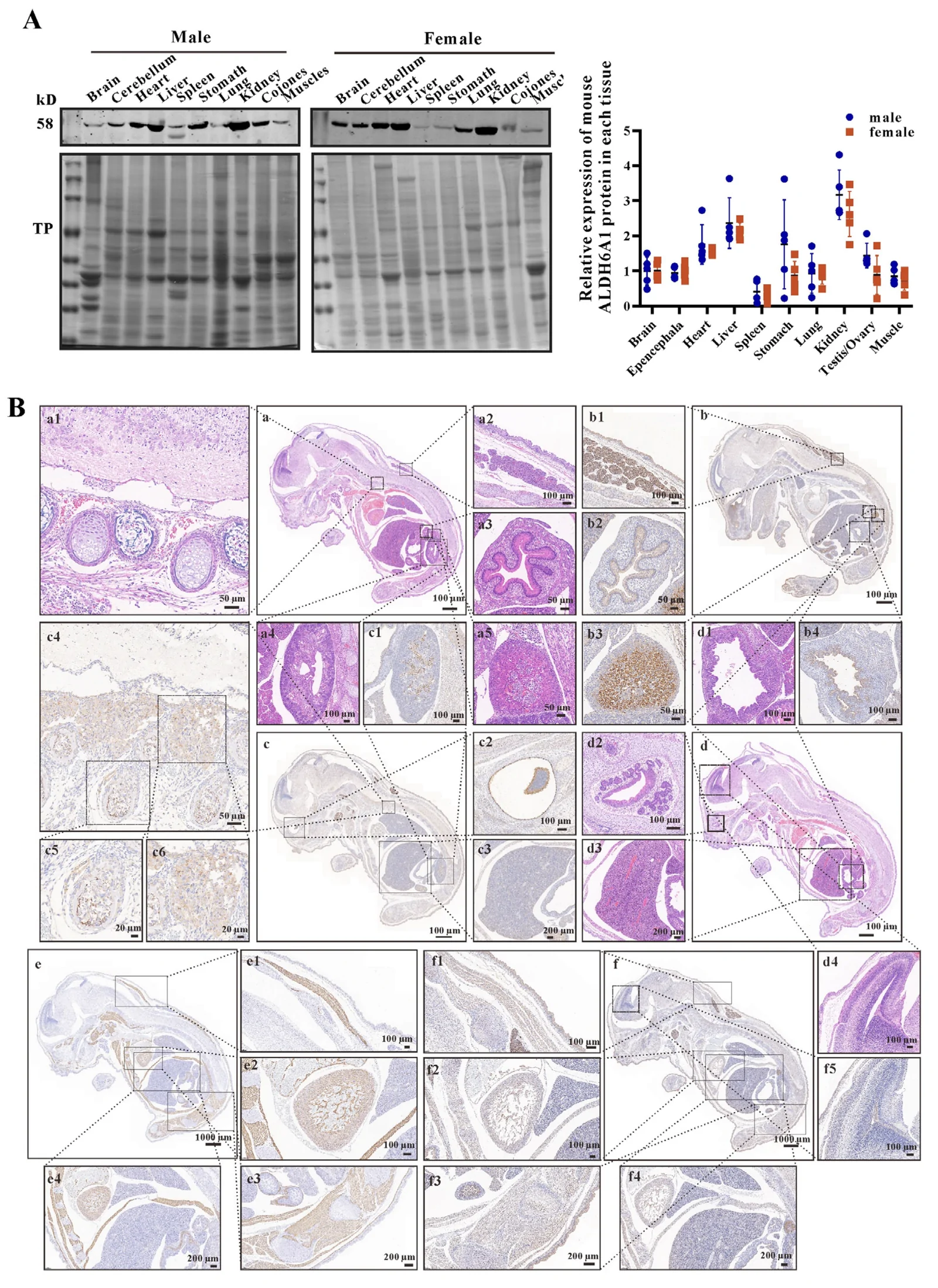
The expression and distribution of ALDH6A1 in adult mice and embryos. (**A**) The expression of ALDH6A1 in various tissues of adult female and male mice was detected using WB. *n* = 5. The total protein was stained with the Coomassie two-band method as the internal reference. (**B**) Sagittal paraffin sections of fetal mice were subjected to H&E staining (**a**,**d**) or immunohistochemistry against ALDH6A1 (**b**,**c**) or DESMIN (**e**,**f**). Whole-section scans are presented in (**a**–**f**), with magnified views of specific tissues and structures indicated by numbered panels. (**a1**,**c4**–**c6**) show magnified views of the spinal cord region at the transverse plane, with (**c5**) highlighting the intervertebral cartilage and (**c6**) the dorsal root ganglion. (**a2**,**b1**) depict the developing lymph node; (**a3**,**b2**), the stomach; (**a4**,**c1**), the kidney; (**a5**,**b3**), the pancreas; (**d1**,**b4**), the intestine; (**c2**,**d2**), the eye; (**c3**,**d3**), the liver; (**e1**,**f1**), the dorsal skeletal muscle; (**e2**,**f2**), the heart; (**e3**,**f3**), the hindlimb; (**e4**,**f4**), the diaphragm; and (**d4**,**f5**), the cortical plate and ventricular zone of the brain. Representative images shown in (**B**) were selected from Supplementary Figure S1 as follows: panel (**b2**) corresponds to Supplementary Figure S1(C4); (**b3**) corresponds to Supplementary Figure S1(B4); (**c1**) corresponds to Supplementary Figure S1(D1); and (**c2**) corresponds to Supplementary Figure S1(A1). Supplementary Figure S1 comprises sections from five different fetal mice.

To systematically assess the expression profile of ALDH6A1 in mammals, we performed Western blot analysis across 11 tissues (brain, heart, liver, spleen, lung, kidney, stomach, intestine, colon, muscle, and gonadal) from wild-type mice (8 weeks old, 5 males and 5 females). Total protein loading was normalized using Coomassie brilliant blue staining to account for tissue specific background. ALDH6A1 was ubiquitously expressed, with the highest levels in kidney and liver; moderate expression was observed in heart, brain, muscle, stomach, intestine, and gonads; spleen and lung displayed comparatively weaker signals (Figure 4A). Quantification confirmed kidney and liver as peak expression organs with no significant sex-dependent differences. This tissue distribution mirrors the human ALDH6A1 expression atlas, supporting the physiological relevance of the mouse model.

To determine the spatiotemporal expression pattern of ALDH6A1 during embryonic development, we performed immunohistochemistry on sagittal paraffin sections of E16.5 mouse embryos. Adjacent sections were stained with hematoxylin and eosin (H&E) for anatomical reference. Low-magnification survey revealed heterogeneous, multi-tissue expression, with marked variations in signal intensity across organs. In the nervous system, spinal cross-sections showed weak to moderate immunoreactivity in vertebral cartilage, whereas intense cytoplasmic staining was observed in dorsal root ganglion (DRG) neurons (Figure 4B(a1,c4–c6). In the telencephalon, the cortical plate was negative, the ventricular zone was distinctly positive, and the intermediate zone was weak to moderate (Figure 4B(d4,f5)). This pattern suggests preferential expression of ALDH6A1 in neural progenitors rather than mature neurons. In the visual system, the developing eye displayed extremely strong, continuous positivity in the retinal pigment epithelium (RPE). Moderate to strong immunoreactivity was also observed in the neural retina, iris/ciliary body epithelium, and corneal/eyelid epithelium, whereas the lens epithelium and lens capsule showed weak staining. The inner layer of the optic cup was centrally negative, with moderate signal restricted to the outer proliferative zone. The extraocular mesenchyme and vitreous cavity were negative (Figure 4B(c2,d2), Supplementary Figure S1(A1–A5)). This pattern of high expression in metabolically active epithelial components of the eye suggests that ALDH6A1 may play an important role in RPE function maintenance and early development of the neural retina. In the musculoskeletal system, skeletal muscle, cardiac muscle, and diaphragm exhibited moderate immunoreactivity that colocalized with DESMIN (Figure 4B(e1–e4,f1–f4)). In the digestive system, gastric and intestinal epithelia were strongly positive (Figure 4B(a3,b2,d1,b4), Supplementary Figure S1(C1–C4)). The pancreas displayed intense diffuse cytoplasmic staining throughout acinar cell, implying a role in pancreatic exocrine function (Figure 4B(a5,b3), Supplementary Figure S1(B1–B4)). In the kidney, strong positivity was localized to developing renal tubule epithelia, appearing as deep brown granular cytoplasmic staining, whereas glomerular regions and surrounding mesenchyme showed weak or no signal (Figure 4B(a4,c1), Supplementary Figure S1(D1–D4)). Additionally, in the dorsal subcutaneous regions, we identified lobulated clusters of intensely positive cells as developing lymph nodes based on their location, morphology, and cellular arrangement, implying a potential role for ALDH6A1 in lymphocyte development or immune responses (Figure 4B(a2,b1)). Notably, the fetal liver exhibited relatively weak and heterogeneous staining, with hepatocyte cytoplasm showing only weak to moderate positivity (Figure 4B(c3,d3), Supplementary Figure S1(E1–E5)). This contrasted with the high hepatic expression observed in adult mice and zebrafish in situ hybridization. We attribute this discrepancy to developmental stage specificity: at the E14–E18, the mouse liver is predominantly hematopoietic with immature metabolic function, and ALDH6A1 upregulation likely accompanies postnatal hepatic metabolic maturation. Together, these data reveal three defining features of ALDH6A1 embryonic expression: tissue ubiquity, developmental dynamics, and cell-type specificity. These features align closely with the multi-system involvement observed in MMSDD patients, supporting the utility of mice as a mammalian validation system for this disorder.

### 3.5. Aldh6a1 Deficiency Causes Dose-Dependent Developmental Defects in Zebrafish Embryos

In eukaryotes, gene transcription produces unprocessed pre-mRNA, whose maturation relies on spliceosome-mediated recognition of conserved splice donor and acceptor sequences. Disruption of these consensus sites causes aberrant splicing, frequently resulting in frameshift mutations and premature translational termination. Splice-blocking morpholino oligonucleotides (spl-MOs) exploit this principle: an antisense oligomer complementary to a splice donor or acceptor site hybridizes with the target pre-mRNA and sterically obstructs spliceosome assembly, thereby interfering with correct splicing and reducing functional protein expression. To assess the impact of Aldh6a1 loss-of-function on vertebrate embryonic development and evaluate zebrafish as a loss-of-function experimental system for MMSDD, we microinjected spl-MO targeting zebrafish *aldh6a1*, with rescue groups co-injected with in vitro transcribed zebrafish *aldh6a1* mRNA to verify phenotype specificity and exclude morpholino-induced nonspecific toxicity. Five experimental groups were established: MO-Ctrl, MO-*aldh6a1*-0.3 mM (low-dose), MO-*aldh6a1*-0.5 mM (high-dose), MO-*aldh6a1*-0.3 mM + *aldh6a1* mRNA, and MO-*aldh6a1*-0.5 mM + *aldh6a1* mRNA. Embryos were monitored and quantified for survival up to 80 hpf, hatching rate at 48 hpf, and malformation rate at 72 hpf.

**Figure 5:**
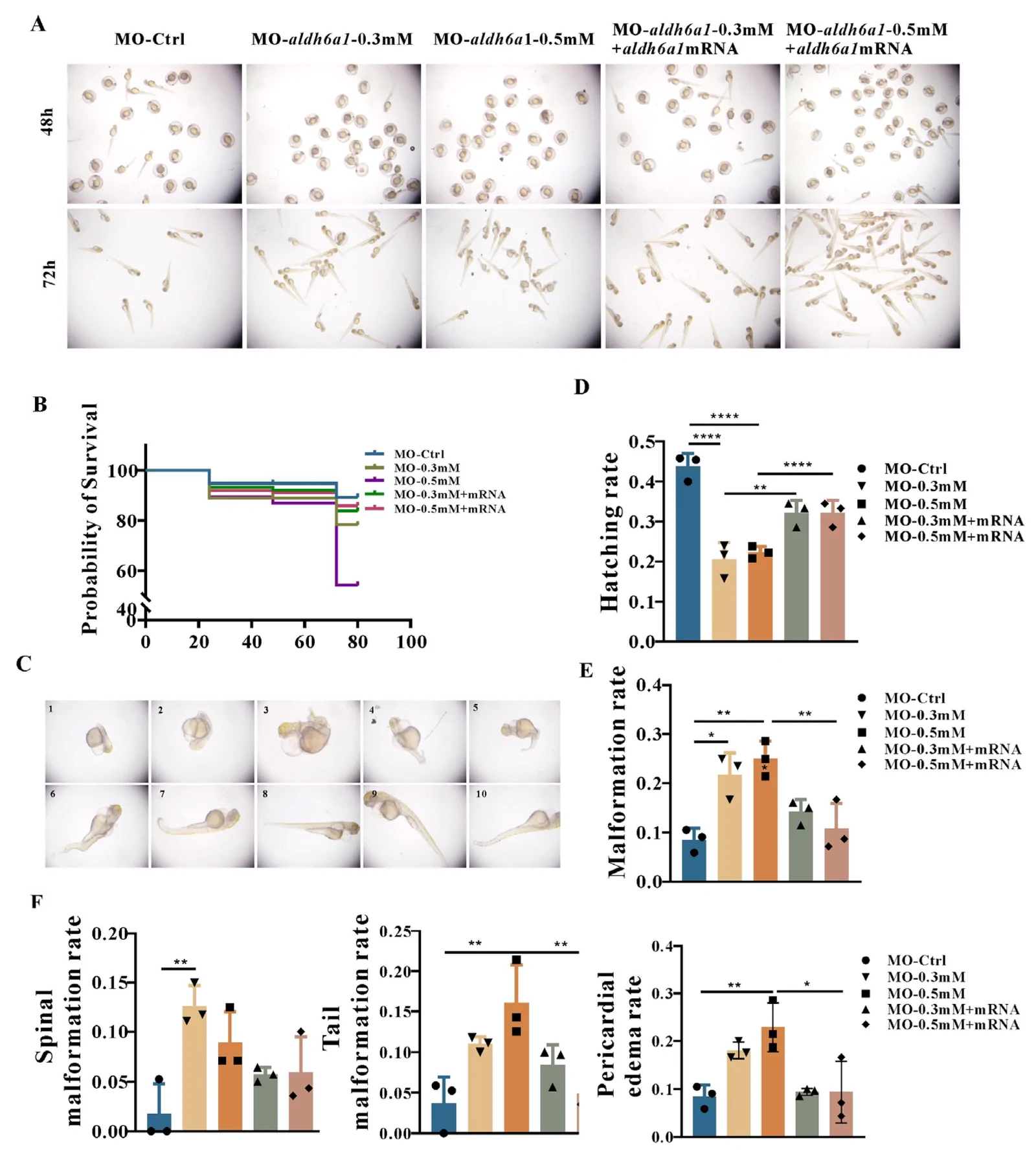
Morpholino-mediated knockdown of *aldh6a1* induces dose-dependent developmental defects in zebrafish. (**A**) Representative bright-field images of zebrafish embryos at 48 and 72 hpf following microinjection with control morpholino (MO-Ctrl), low-dose (0.3 mM) or high-dose (0.5 mM) *aldh6a1* splice-blocking morpholino (MO-*aldh6a1*), and corresponding rescue groups co-injected with *aldh6a1* mRNA. (**B**) Kaplan–Meier survival curves of zebrafish embryos up to 80 hpf. (**C**) Representative images of individual embryos at 72 hpf illustrating specific malformation phenotypes: 1 tail development failure; 2 tail development failure with pericardial edema; 3 scoliosis; 4 scoliosis with curled tail; 5 scoliosis with curled tail and pericardial edema; 6 scoliosis with curled tail, pericardial edema, and developmental retardation; 7 mild tail curling; 8 delayed yolk sac absorption; 9 delayed yolk sac absorption; 10 mild tail curling with delayed yolk sac absorption. (**D**) Quantification of normalized hatching rate at 48 hpf. (**E**) Quantification of total normalized malformation rate at 72 hpf. (**F**) Dissection of specific malformation types at 72 hpf: spinal curvature (**left**), tail curling (**middle**), and pericardial edema (**right**). Data are presented as mean ± SD. Statistical significance was determined by one-way ANOVA with Tukey’s post hoc test (* *p* < 0.05, ** *p* < 0.01, **** *p* < 0.0001).

Morphological examination revealed that most control embryos developed normally with straight body axes, regular heartbeats, and efficient yolk sac absorption. In contrast, knockdown embryos displayed dose-dependent increases in phenotypic abnormalities: the low-dose group (0.3 mM) showed a subset of embryos with mild body axis curvature and delayed yolk sac absorption (Figure 5C(7–10)), whereas the high-dose group (0.5 mM) displayed a markedly higher proportion of severe phenotypes including scoliosis, curled tail, pericardial edema, and occasional tail truncation (Figure 5C(1–6)), with some embryos dying before 72 hpf (Figure 5A). These malformation phenotypes closely mirror MMSDD clinical features: spinal curvature corresponds to skeletal abnormalities, curled tail and tail truncation parallel hypotonia and motor deficits, pericardial edema indicates cardiovascular involvement, and overall developmental delay aligns with the growth retardation observed in affected individuals. Survival analysis (Figure 5B) revealed that control embryos maintained 90% survival at 80 hpf, whereas low-dose and high-dose knockdown groups dropped to approximately 80% and 55%, respectively. Rescue groups recovered to approximately 85% survival, significantly higher than their corresponding knockdown counterparts, confirming that the lethal phenotype arises from Aldh6a1-specific deficiency rather than MO non-specific toxicity.

Hatching rate quantification (Figure 5D) demonstrated that control embryos achieved 45% hatching at 48 hpf, while low-dose and high-dose knockdown groups fell to approximately 21% and 25%, respectively. Rescue groups recovered to approximately 35% and 33% hatching rates, significantly higher than matched knockdown groups, indicating that Aldh6a1 loss causes gene-specific hatching impairment amenable to partial rescue by exogenous mRNA. Detailed malformation analysis further revealed the dose-dependent nature and rescue efficacy of these phenotypes (Figure 5E,F). Total malformation rate increased from near-baseline in controls to approximately 2.5-fold and 3.0-fold in low-dose and high-dose knockdown groups, respectively, with rescue groups declining to approximately 1.6-fold and 1.3-fold (Figure 5E), significantly below their matched knockdowns. Dissection of specific malformation types showed that spinal curvature rate rose approximately 7.0-fold and 4.0-fold in low-dose and high-dose groups, respectively, with rescue groups falling approximately 3.0-fold and 3.5-fold; tail curling rate increased approximately 3.0-fold and 4.5-fold, with rescue groups recovering to approximately 2.0-fold and 1.2-fold; and pericardial edema rate elevated approximately 1.8-fold and 2.2-fold (Figure 5F), with rescue groups returning to near-control levels. Notably, rescue groups showed significant improvement without complete restoration, which may reflect limitations in mRNA dosage, stability, or translational efficiency, or alternatively suggest partial functional redundancy or compensatory mechanisms.

Collectively, these data demonstrate that Aldh6a1 deficiency elicits dose-dependent, multi-system developmental defects in zebrafish, encompassing the nervous system, cardiovascular system, and overall developmental progression, all partially reversible by exogenous *aldh6a1* mRNA rescue. This loss-of-function model not only establishes the essential requirement of Aldh6a1for vertebrate early development at the causal level but also provides a tractable in vivo platform for investigating MMSDD pathogenesis. Given the optical transparency, rapid development, and high-throughput amenability of zebrafish embryos, this model holds promise as a preclinical platform for screening Aldh6a1 enzymatic modulators, pharmacological chaperones, and potential gene therapy strategies.

### 3.6. Cross-Species Concordance of ALDH6A1/Aldh6a1 Expression Supports Complementary MMSDD Experimental System

To systematically evaluate the concordance between zebrafish and mouse experimental findings, we aligned the developmental stages, detection methods, and anatomical systems assessed in both species with the clinical manifestations reported in MMSDD patients. As summarized in Table S1, ALDH6A1/Aldh6a1 exhibited conserved expression across both experimental systems in the nervous system (neural tube/brain in zebrafish; DRG, cortical ventricular zone, and neural retina in mice), musculature (somites/myotomes in zebrafish; skeletal, cardiac, and diaphragm muscles in mice), liver (hepatic primordium in zebrafish; fetal and adult hepatocytes in mice), and gastrointestinal tract (intestine in zebrafish; gastric/intestinal epithelium and pancreas in mice). Mitochondrial targeting of ALDH6A1 orthologs was similarly conserved across human, mouse, and zebrafish proteins. These expression domains directly correspond to the primary lesion sites observed in MMSDD patients, including the central nervous system, skeletal muscle, liver, and digestive organs. Collectively, this cross-species alignment supports the validity of zebrafish and mice as complementary experimental systems for investigating MMSDD pathogenesis.

## 4. Discussion

Methylmalonate semialdehyde dehydrogenase deficiency (MMSDD) is an extremely rare autosomal recessive disorder caused by mutations in the *ALDH6A1* gene [2]. Since its initial description by Pollitt et al. in 1985, only five cases have been reported worldwide [2]. MMSDD exhibits marked clinical heterogeneity; core features include growth retardation, hypomyelination, and hypotonia, while variable manifestations encompass visual impairment, gastroesophageal reflux, microphthalmia, hepatosplenomegaly, and additional systemic manifestations [1,2,3,4,5]. At the biochemical level, affected individuals exhibit marked accumulation of ALDH6A1-associated metabolites, including 3-hydroxyisobutyric acid, methylmalonic acid, and methylmalonate semialdehyde (MMSA), in urinary organic acid profiles. Owing to this phenotypic heterogeneity and the extreme scarcity of cases, diagnosis of MMSDD relies almost exclusively on genetic sequencing, while the underlying pathogenic mechanisms and therapeutic strategies remain largely unexplored. In this study, we established a four-tiered framework; cross-species conservation (Figure 1), subcellular localization (Figure 2), spatiotemporal expression profiling (Figure 3 and Figure 4), and loss-of-function rescue (Figure 5) in order to systematically validate mouse and zebrafish as faithful preclinical experimental systems for MMSDD. ALDH6A1/Aldh6a1 exhibits remarkable conservation across these species at the levels of gene architecture, protein sequence, three-dimensional structure, and mitochondrial targeting. The dynamic embryonic expression pattern and adult tissue distribution of ALDH6A1/Aldh6a1 closely mirror the multi-system involvement observed in MMSDD patients. Furthermore, the dose-dependent developmental defects elicited by *aldh6a1* knockdown in zebrafish are substantially rescued by exogenous mRNA, confirming the specificity of these phenotypes. Collectively, these findings establish mouse and zebrafish as complementary preclinical platforms: murine expression profiling is particularly suited for metabolic phenotyping and therapeutic intervention studies, whereas the zebrafish knockdown system offers a tractable framework for high-throughput drug screening and developmental mechanistic investigation.

The evolutionary conservation of ALDH6A1/Aldh6a1 across vertebrates provides the foundation for the cross-species modeling strategy used in this study. The preservation of gene architecture, chromosomal synteny, and all four functional domains from fish to mammals indicates that ALDH6A1/Aldh6a1has undergone stringent purifying selection, reflecting its non-redundant role in mitochondrial metabolism (Figure 1). For ultra-rare disorders such as MMSDD, where patient material is scarce, this deep molecular and subcellular fidelity justifies the use of zebrafish and mice to dissect pathogenic mechanisms.

The expression pattern of ALDH6A1 directly determines the spectrum of organs affected by its mutations and the developmental time window of injury. Using zebrafish in situ hybridization and mouse embryonic immunohistochemistry, we characterized the ALDH6A1/Aldh6a1 expression lineages in relation to the clinical phenotypes of MMSDD patients. During early somitogenesis (4–15 somites), zebrafish *aldh6a1* transcripts are restricted to the neural plate and subsequently the neural tube, forming a linear domain along the anterior–posterior axis. This pattern suggests a role in neural tube formation, maintenance of neural progenitors, or early neuronal differentiation [33,34,35]. Hypomyelination is a prominent feature of MMSDD, occurring after CNS regionalization [36]. The early enrichment of *aldh6a1* in the neural tube suggests that Aldh6a1may influence subsequent myelination, potentially through effects on oligodendrocyte differentiation or myelin-related signaling [37]. Consistent with this, disruptions in aldehyde metabolism have been shown to impair neural development and myelination [38,39]. From 24–48 hpf, *aldh6a1* mRNA expression expands beyond the neuroectoderm while persisting in the CNS. Concurrently, periodic, segmented stripe-like patterns emerge in somites and progressively refine, suggesting a role for Aldh6a1 in myogenesis. BCAA metabolism is essential for skeletal muscle energy supply [40,41], and ALDH6A1 participates in distal BCAA catabolism. Accordingly, its deficiency may impair myocellular energy metabolism, affecting myofiber differentiation and maturation, thereby potentially contributing to the hypotonia and growth retardation observed in MMSDD. Furthermore, as mesoderm-derived structures, somites play a critical role in organizing adjacent neural crest cell migration and differentiation [42,43,44,45]; defects in somitogenesis may therefore indirectly impair neural crest development, exacerbating neurological abnormalities [46]. Notably, *aldh6a1* mRNAlevels remain relatively stable between 24 and 48 hpf, a critical developmental window during which the zebrafish CNS is rapidly established and somites differentiate into mature myomeres [47,48,49]. This sustained expression suggests that Aldh6a1 functions as a metabolic housekeeping factor, providing essential basal support rather than acting as a transiently induced regulatory protein. Both neurogenesis and myogenesis are highly energy-demanding processes that rely on robust mitochondrial function and amino acid metabolism [50,51]. As a key enzyme in valine catabolism, Aldh6a1 likely ensures a continuous supply of metabolic substrates and efficient clearance of toxic intermediates, thereby maintaining the metabolic homeostasis required for proper neural and muscular morphogenesis. Accordingly, loss of Aldh6a1 function may compromise this metabolic support, leading to impaired neural and muscle development, consistent with the hypomyelination and hypotonia observed in MMSDD patients. As zebrafish development progresses, *aldh6a1* mRNA expression becomes evident in the liver primordium and intestine at 72 hpf, appearing as clustered hepatic signals and linear intestinal stripes. After 96 hpf, expression in both tissues intensifies markedly, coinciding with peak liver maturation and intestinal differentiation. During this stage, the liver undergoes rapid hepatocyte proliferation and metabolic maturation, while the intestine develops epithelial folding, villus formation, and digestive-absorptive functions—processes highly dependent on energy metabolism [52,53,54,55]. Liver contributes importantly to downstream branched-chain keto acid oxidation and systemic metabolic regulation [56]. Elevated Aldh6a1 expression reflects its role in hepatocyte mitochondrial metabolism, where it catalyzes the terminal catabolic steps of valine and isoleucine, thereby ensuring efficient conversion of these amino acids into energy substrates and preventing toxic intermediate accumulation. Concurrently, prominent intestinal Aldh6a1 expression indicates a role in digestive organ development, likely by sustaining energy demands for epithelial proliferation, differentiation, and barrier formation. We speculate that Aldh6a1 deficiency compromises mitochondrial ATP production in esophageal and gastrointestinal smooth muscle, diminishing the energy supply required for actin–myosin cross-bridge cycling and Ca^2+^-ATPase-mediated calcium handling; this deficit impairs resting sphincter tone and peristaltic coordination, thereby permitting reflux. Accumulated reactive aldehyde intermediates may further damage contractile proteins through adduct formation, aggravating motility dysfunction. Alternatively, reflux may represent an extension of the generalized hypotonia characteristic of MMSDD, reflecting systemic muscular impairment rather than a primary enteric defect. Another possibility is that early Aldh6a1 deficiency disrupts vagal neural crest-derived enteric nervous system development, given its strong neural tube expression, thereby causing secondary gastrointestinal dysmotility independent of direct smooth muscle metabolic insult. In addition, the direct expression of Aldh6a1 in the intestinal primordium raises the possibility that its loss may impair epithelial energy metabolism or barrier integrity. Barrier disruption increases permeability, promoting bacterial translocation and local inflammation, potentially compromising gastrointestinal motility and exacerbating reflux. These findings suggest a possible molecular link between developmental ALDH6A1 expression and gastrointestinal phenotypes associated with MMSDD.

These murine studies provided complementary validation of these findings from a mammalian perspective. In adult mice, ALDH6A1 was most abundantly expressed in the kidney and liver, characterized by high metabolic demand and active amino acid metabolism [57]. The distribution pattern largely mirrored that in humans, supporting the utility of these murine data for understanding ALDH6A1 function. Strong ALDH6A1 expression was also detected in metabolically active ocular epithelia, particularly the retinal pigment epithelium (RPE), suggesting involvement in retinal energy metabolism and neural retina development. Expression in the corneal/eyelid and lens epithelia further indicates roles in ocular surface barrier maintenance and lens transparency. These findings provide a mechanistic basis for understanding visual impairments in MMSDD. In contrast to robust hepatic expression in adult mice and strong zebrafish in situ hybridization signals, fetal mouse liver showed weak, heterogeneous ALDH6A1 staining. This developmental discrepancy reflects the predominantly hematopoietic function of E14–E18 mouse liver, with high ALDH6A1 expression likely accompanying postnatal metabolic maturation. Consequently, hepatic metabolic abnormalities in MMSDD may be age-dependent or progressive.

Zebrafish *aldh6a1* knockdown produced dose-dependent, multi-system developmental defects, including spinal curvature, tail coiling, pericardial edema, and reduced survival. These phenotypes closely mirrored key clinical features of MMSDD, notably skeletal abnormalities, hypotonia, and cardiovascular involvement. Importantly, rescue experiments demonstrated that these abnormalities were directly attributable to Aldh6a1 loss of function, thereby excluding morpholino-induced nonspecific toxicity. Together, these findings establish ALDH6A1/Aldh6a1 as a critical determinant of early vertebrate development and validate zebrafish as a robust in vivo model of MMSDD. Leveraging the optical transparency, rapid embryogenesis, and genetic tractability of zebrafish, this system provides a scalable platform for high-throughput interrogation of disease mechanisms and systematic screening of therapeutic candidates. In parallel, the mouse expression analysis affords mammalian validation and enables precise spatial and tissue-specific characterization of ALDH6A1 expression in relation to disease-relevant phenotypes.

Taken together, these complementary experimental systems define a cross-species functional framework for ALDH6A1. The zebrafish model enables rapid, high-resolution dissection of developmental and cellular mechanisms, whereas murine expression profiling supports in-depth metabolic and organ-level phenotyping. This integrated strategy addresses a central challenge in rare disease research—the limited availability of patient material—by bridging molecular, cellular, and organismal scales. Consequently, it provides a coherent mechanistic basis for the multi-system heterogeneity associated with *ALDH6A1* mutations and establishes a preclinical platform for the development of precision therapeutic strategies in MMSDD.

## 5. Conclusions

This study establishes a cross-species experimental framework for investigating MMSDD by systematically characterizing ALDH6A1/Aldh6a1 conservation, subcellular localization, spatiotemporal expression, and loss-of-function phenotypes in zebrafish and mice. Our findings demonstrate that ALDH6A1/Aldh6a1 is evolutionarily conserved from fish to mammals and is essential for mitochondrial metabolism during neurogenesis, myogenesis, and organogenesis. The complementary use of zebrafish knockdown experiments and murine expression profiling provides mechanistic insight into the multi-system heterogeneity of MMSDD and identifies tractable preclinical platforms for therapeutic discovery. Future studies utilizing *Aldh6a1* knockout mouse models and patient-derived induced pluripotent stem cells will be essential to validate the proposed metabolic mechanisms and advance precision intervention strategies for this ultra-rare disorder.

## Data Availability

The datasets generated during the current study are available from the corresponding author upon reasonable request.

## Supplementary Materials

The following supporting information can be downloaded at https://www.mdpi.com/article/10.3390/biology15161345/s1, Figure S1: Immunohistochemical staining of ALDH6A1in various tissues of mouse fetuses. (A1–A5) Eye region; (B1–B4) pancreas; (C1–C4) stomach; (D1–D4) kidney; (E1–E5) liver. Scale bars are indicated in each panel; Table S1: Cross-species comparison of ALDH6A1/Aldh6a1 expression, subcellular localization, and functional validation relative to MMSDD clinical manifestations.
