# Supplementary material for: Cross-Species Conservation and Function of ALDH6A1/Aldh6a1 Validate Zebrafish and Mouse as Complementary Experimental Systems for Methylmalonate Semialdehyde Dehydrogenase Deficiency
Source: Biology (Basel). 2026 Aug 8;15(16):1345. doi: 10.3390/biology15161345 (PMC13509631; doi:10.3390/biology15161345)
Supplement: Supplementary file 1 [file biology-15-01345-s001.zip › biology-4451952-supplementary.pdf]

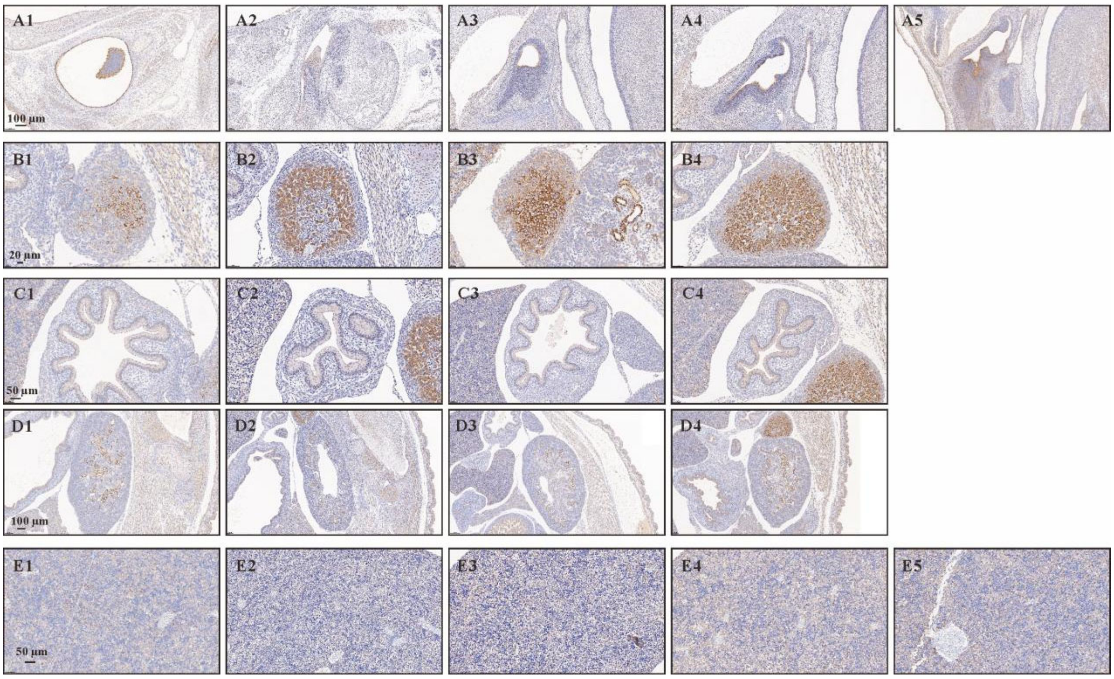

Supplementary Figure S1. Immunohistochemical staining of ALDH6A1in various tissues of mouse fetuses. (A1–A5) Eye region; (B1–B4) pancreas; (C1–C4) stomach; (D1–D4) kidney; (E1–E5) liver. Scale bars are indicated in each panel.

Table S1 Cross-species comparison of ALDH6A1/Aldh6a1 expression, subcellular localization, and functional validation relative to MMSDD clinical manifestations.

	**Human MMSDD Phenotype**	**Zebrafish (*Danio rerio*)**	**Mouse (*Mus musculus*)**	**Cross-species Concordance**
**Nervous system**	Hypomyelination, developmental delay	Neural tube, brain (WISH, 4–96 hpf)	DRG, cortical VZ, neural retina (IHC, E16.5)	Conserved CNS expression
**Muscle**	Hypotonia	Somites, myotomes (WISH)	Skeletal/cardiac muscle, diaphragm (IHC + Desmin)	Conserved myogenic expression
**Liver**	Hepatosplenomegaly	Liver primordium (WISH, 72–96 hpf)	Hepatocytes (IHC, weak at E16.5; high in adult WB)	Developmental stage-specific maturation

**Eye**	Visual impairment, microphthalmia	——	RPE, neural retina, corneal epithelium (IHC)	Mammalian-specific validation
**Gastrointestinal**	Gastroesophageal reflux	Intestine (WISH, 72–96 hpf)	Gastric/intestinal epithelium, pancreas (IHC)	Conserved digestive expression
**Subcellular localization**	Intracellular aldehyde accumulation	Mitochondria (IF, HEK293T)	Mitochondria (IF, HEK293T)	Conserved mitochondrial targeting
**Functional validation**	Recessive metabolic defect	MO-knockdown: spinal curvature, edema, survival defect (rescue confirmed)	——	Zebrafish-only functional data; mouse expression corroboration
